# Use of Oral Health Impact Profile-14 (OHIP-14) in Different Contexts. What Is Being Measured?

**DOI:** 10.3390/ijerph182413412

**Published:** 2021-12-20

**Authors:** Lucas Arrais Campos, Timo Peltomäki, João Marôco, Juliana Alvares Duarte Bonini Campos

**Affiliations:** 1 Faculty of Medicine and Health Technology, Tampere University, 33520 Tampere, Finland; timo.peltomaki@tuni.fi; 2 School of Dentistry of Araraquara, São Paulo State University (UNESP), São Paulo 14801-385, Brazil; 3 Faculty of Health Sciences, Institute of Dentistry, University of Eastern Finland, 70211 Kuopio, Finland; 4 Department of Oral and Maxillofacial Diseases, Kuopio University Hospital, 70210 Kuopio, Finland; 5 Department of Ear and Oral Diseases, Tampere University Hospital, 33520 Tampere, Finland; 6 William James Center for Research (WJCR), University Institute of Psychological, Social and Life Sciences (ISPA), 1100-304 Lisbon, Portugal; jpmaroco@ispa.pt; 7 School of Pharmaceutical Sciences of Araraquara, São Paulo State University (UNESP), Araraquara 14801-902, Brazil; juliana.campos@unesp.br

**Keywords:** psychometrics, oral health, validation study

## Abstract

The Oral Health Impact Profile-14 (OHIP-14) has been used to assess the impact that oral health problems can have on an individual’s life. Different theoretical models were proposed to evaluate the results. The aims of this study were to evaluate the fit of different factorial models of the OHIP-14 to non-dental patients (NDP) and dental patients (DP) samples from Brazil and Finland and to estimate the differential functioning of the items in the OHIP-14 between the samples. Two studies were conducted, one in Brazil and the other in Finland, composed of five samples (Brazil—Sample 1 (S1): DP, *n* = 434, age: 25.3 [*SD* = 6.3] years; S2: NDP, *n* = 1486, age: 24.7 [*SD* = 5.6] years; S3: DP, *n* = 439, age: 29.0 [*SD* = 6.7] years; Finland—S4: DP, *n* = 482, age: 26.3 [*SD* = 5.4] years; S5: NDP, *n* = 2425, age: 26.7 [DP = 5.5] years). The fit of the OHIP-14 models to the data was estimated using a confirmatory strategy (validity based on the internal structure). Differential item functioning (DIF) between samples was estimated. For NDP from both countries, the response pattern severely violated the normality assumption in six items of the OHIP-14, indicating that the instrument does not fit for these samples. For DP, the model with the best fit was unifactorial, which deals with the estimation of the general impact of oral health on an individual’s life, without addressing specific dimensions. Configural invariance was refuted between samples. DIF indicated that the characteristic of the sample (NDP and DP) in both countries interfered in the response given to the items, with the response level being more adequate for the latent PD trait. The validity of data related to the impact of oral health problems on an individual’s life was confirmed through a unifactorial model. OHIP-14 works properly in DP samples and was limited in NDP samples, being also influenced by cultural context and age.

## 1. Introduction

Oral health-related quality of life (OHRQoL) is a multidimensional concept that involves biopsychosocial aspects related to oral health [1] and is based on the World Health Organization definition that considers health as the state of complete physical, mental and social well-being. Nevertheless, OHRQoL is commonly viewed from a reductionist perspective, in which only the individual’s own perception is considered, not including biopsychosocial aspects. Most studies describe OHRQoL as the impact of orofacial conditions and dental treatments perceived by the individual [2,3]. This is the definition adopted in this study.

Although the OHRQoL by itself does not reveal the clinical oral status, this perspective allows the identification of an individual’s perception of oral health and its relevance and impact on their life. For research, this relevance lies in the possibility that the OHRQoL represents an indicator for public health [3], which may be a guide both in relation to the limitations of oral health of populations and the impact of oral health and/or dental treatments on people’s lives. In clinical practice, information about OHRQoL allows the development of a patient-centered treatment plan. For education, OHRQoL allows health professionals, graduated or in training, to see their patient not only as an aspect and/or physical problem to be treated, but as a human being inserted into unique life contexts and whose involvement in health is perceived in a unique way. All these points contribute to the construction of more humane evidence-based dentistry focused not only on technique and/or disease elimination, but also on health promotion. However, as it is a concept that is not directly measurable (latent), evaluating the OHRQoL can be a challenge.

To measure OHRQoL, specific instruments are used, such as the Geriatric Oral Health Assessment Index (GOHAI) [4], Oral Impacts on Daily Performances (OIDP) [5], and the Oral Health Impact Profile (OHIP) [6,7]. The latter is the most widely used both by researchers and clinicians [8]. The OHIP was originally developed by Slade and Spencer [6] in Australia, containing 49 items (OHIP-49) formulated from statements obtained in interviews with dental patients. These items were distributed considering seven dimensions (functional limitation, physical pain, psychological discomfort, physical disability, psychological disability, social disability, and handicap) elaborated from the theoretical model proposed by Locker [9]. Shortened versions of this instrument were developed, highlighting the Oral Health Impact Profile-14 (OHIP-14) [7].

A reduction of items was performed using statistical procedures on sample data from Australian individuals aged over 60 years [5]. Since its publication, the OHIP-14 has been translated and adapted into different languages [10,11,12,13,14]. It has also been widely applied in different samples and contexts different from those in which the instrument was proposed and evaluated, for example, in the general population (non-patient) [12,13,15], young orthodontic patients [11], indigenous population [15] and postpartum women [10,16].

However, as OHIP-14 is an instrument for measuring a latent concept, it is necessary to ensure that it really can measure what it was proposed to measure, that is, the perception of the impact that an oral health problem can have on an individual’s life. Previous use of the instrument in different samples is not able to guarantee this. Therefore, it seems necessary to carry out analytical analyses capable of attesting to the validity of data obtained in different samples and/or contexts using the instrument.

Although two studies, one using a sample of the general population of British adults [17] and the other Brazilian dental patients [18], confirmed the adequacy of the original factorial structure of OHIP-14, other studies have refuted that OHIP (full or shortened version) has this factorial structure [12,13,15,16,19]. Montero et al. [13] found a trifactorial structure of the OHIP (psychosocial impacts, pain-discomfort and functional limitation) in a sample of Spanish workers who were undergoing routine medical examinations at an employment risk prevention center. Santos et al. [16] found unifactorial structure in Brazilian samples of postpartum women and elderly individuals, including individuals with and without oral health impairment. However, it is worth noting that most of these studies used the OHIP-14 in a different context and population from those in which the instrument was elaborated, paving the way for questioning whether this instrument can preserve the latent concept that it should measure.

Given the different proposals for factorial structures for OHIP-14 and knowing that its fitting may be influenced by culture, study population (e.g.,: dental patient or non-dental patient) and other individual and clinical characteristics, the present study was conducted to build evidence concerning the measurement validity of the OHIP-14 when applied to different populations from different countries. The aims of the present study were to evaluate the fit of different factorial models of the OHIP-14 (seven factors, three-factor and one-factor) to different samples (non-dental patient and dental patient) from Brazil and Finland and to estimate the differential functioning of the items in the OHIP-14 in the non-dental patient and dental patient samples.

## 2. Methods

To address the aims, two studies were conducted independently, one in Brazil (Study 1) and one in Finland (Study 2). The possibility to conduct studies in countries with distinct cultural characteristics is interesting, as it has the potential to strengthen the evidence of OHIP-14’s functionality and the validity and reliability of the obtained data. It should be clarified that the selection of Brazil and Finland for the study was specifically based on the convenience of the researchers whose work is located in these countries. The description of the two studies is found below.

### 2.1. Study Design and Sampling

Both studies were cross-sectional studies with non-probabilistic convenience sampling. Individuals between 18 and 40 years of age were included in the study. Age was limited to 40 years to minimize the effect of this variable on the results.

**Figure 1:**
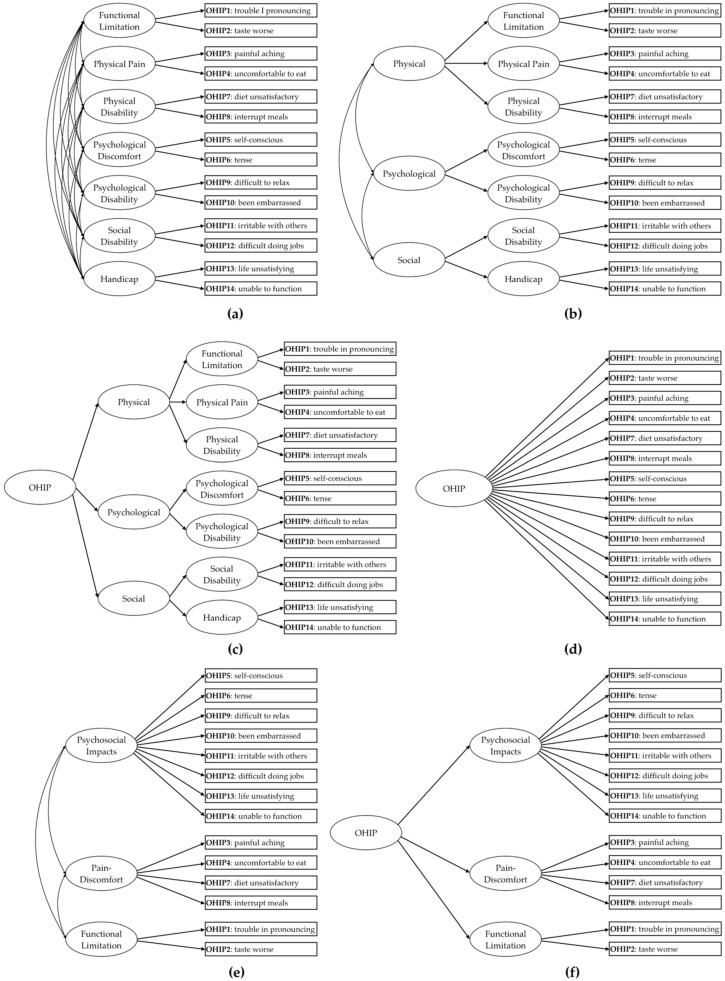
Factorial models of the Oral Health Impact Profile-14 (OHIP-14) tested in the study: (**a**) 7 first-order factors; (**b**) and (**c**) second- and third-order hierarchical models with 7 first-order factors; (**d**) unifactorial model; (**e**) trifactorial model; (**f**) second-order hierarchical model with 3 first-order factors.

The proposal by Hair et al. [20] was adopted to calculate the minimum sample size. The authors recommend a minimum of 5–10 participants per parameter to be tested in the factorial model. Considering that the factorial model with the largest number of parameters tested in this study has 42 parameters (Figure 1a), the minimum sample size is 210–420 individuals. However, because we aimed to estimate psychometric properties and differential item functioning of OHIP-14 for non-dental patients and dental patients, the number of participants should be large enough in each sample.

In addition, a third sample of Brazilian dental patients (Faculty of Dentistry of Araraquara—UNESP) (Sample 3) from our previous study [18] was used in Study 1. To make comparisons between samples, Sample 3 was limited to only patients aged between 18 and 40 years. Despite the delimitation of the age group, the mean age of Sample 3 was higher than that observed in Samples 1 and 2. Table 1 presents general descriptions of the samples.

### 2.2. Procedures and Ethical Aspects

In Study 1, adult patients attending the clinics of the Faculty of Dentistry of Araraquara—UNESP (periodontology, dentistry, emergency, prosthodontics, oral medicine, and surgery clinics) from August 2018 to December 2019 were invited to participate in the study for composing Sample 1. For Sample 2, staff from the same university were invited to participate. After completing the data collection, they were asked to invite their families and colleagues to participate in the study (snowball sampling). The measuring instrument was self-filled using the paper-and-pencil method.

In Study 2, an online survey was used since data collection took place after the onset of the COVID-19 pandemic, which imposed social isolation, making the paper-and-pencil collection strategy as performed in Study 1 unfeasible. For Sample 4 and Sample 5, students and staff from Tampere University and University of Oulu were initially invited to participate via institutional email from June to July 2020. The invitation message described the purpose of the study and had a link to the online measurement instrument. The online form was created using the LimeSurvey program (LimeSurvey GmbH, Hamburg, Germany; http://www.limesurvey.org (accessed on 24 October 2021)) located on the Tampere University server. To identify individuals who were undergoing dental treatment at the time of participation in the study, a question was prominently inserted before accessing the page with the items OHIP-14. The snowball sampling strategy was adopted to recruit more participants. Thus, at the end of the online survey, participants were asked to send the research link to their personal contacts via email and social networks.

Study 1 was approved by the Research Ethics Committee of São Paulo State University (Unesp), School of Dentistry, Araraquara (CAAE: 01040312.5.0000.5416 and 88600318.3.0000.5416). Approval for Study 2 data collection was obtained from the Data Protection Officer at Tampere University, in accordance with the European Union’s General Data Protection Regulation. In both studies, only individuals who gave informed consent participated in the study.

### 2.3. Measuring Instrument

To assess the profile of the impact of oral health on the lives of individuals, the Portuguese and Finnish versions [10,14] of the OHIP-14 [7] were used. This instrument has a 5-point Likert-type response scale (0: never, 1: hardly ever, 2: occasionally, 3: fairly often, 4: very often).

The original factor structure of the OHIP-14 [7] was elaborated following the theoretical model proposed by Locke [9], containing seven first-order factors (dimensions: functional limitation, physical pain, psychological discomfort, physical disability, psychological disability, social disability, and handicap) [7] (Figure 1a). Following this proposal, Zucoloto et al. [18] also showed that it is possible to obtain a second-order hierarchical model (second-order factors: physical, psychological, and social, Figure 1b) and the inclusion of a third-order hierarchical factor (Figure 1c). The unifactorial (Figure 1d) and the first- and second-order trifactorial (psychosocial impacts, pain-discomfort, and functional limitation, Figure 1e,f) models were also tested, as some studies suggest these structures [13,16].

### 2.4. Validity of Data Analysis

The evidence of validity of the data obtained by OHIP-14 in the samples was verified following the proposal of the Standards for Educational and Psychological Testing [21]. Content validity, validity based on internal structure, validity based on response process and consequence validity were considered.

### 2.5. Content Validity

Content validity was initially assessed in each study by a panel of three expert judges (Study 1: native Portuguese experts; Study 2: native Finnish experts). They assessed the grammatical, semantic, and idiomatic terms of the items. They also evaluated whether the content of the items preserves the concept proposed (impact of oral health on an individual’s life) in the English version [6,7] of the instrument and if it suited the context of each country. Then, a pilot study was conducted among the target population of each study, following the same procedures as the definitive study, to assess the Incomprehension Index of the items. This index verifies whether there are difficulties in understanding the content of the items by the participants. Values below 15% were considered indicative that the item is suitable for understanding in the population.

### 2.6. Validity Based on Internal Structure

The OHIP-14 factorial models tested are shown in Figure 1. The validity based on the internal structure was evaluated through factorial, convergent, and discriminant validity. Initially, the psychometric sensitivity of the items was verified through descriptive statistics (mean, median, standard deviation, minimum and maximum values, skewness, and kurtosis) of the answers given by the participants. Absolute values of skewness and kurtosis lower than 3 and 10, respectively, were indicative of non-severe violation of the normal distribution [22], attesting to the psychometric sensitivity of the items [23].

Factorial validity was estimated using confirmatory analysis with the robust Weighted Least Squares Mean and Variance Adjusted (WLSMV) estimation method. The indices used to evaluate the fit of the model to the data were the comparative fit index (CFI), the Tucker–Lewis index (TLI), root mean square error of approximation (RMSEA), and standardized root mean square residual (SRMR) [23,24]. The factorial loadings (λ) of the items were also considered. The fit of the factorial model to the data was considered adequate when CFI and TLI > 0.90, RMSEA < 0.10, SRMR < 0.08, and λ ≥ 0.50 [23,24]. If necessary, the modification indices, estimated by Lagrange Multipliers (LM), were inspected to verify the existence of correlation between item errors (LM > 11) [23].

Convergent validity was evaluated based on the Average Extracted Variance (AVE) [25]. Values of AVE ≥ 0.50 were considered adequate [25] for factorial models with more than one factor, the discriminant validity was also estimated through correlational analysis between the factors [25], being considered adequate if AVE_i_ e AVE_j_ ≥ r_ij_^2^. These analyses were performed in the R program (R Core Team, 2020) using the *lavaan* package [26].

Data reliability (by factor and for each model) was estimated using the ordinal alpha coefficient (α) and composite reliability (CR) [23]. Values of α and CR equal to or greater than 0.70 were considered indicative of adequate reliability [23]. The suggestion of the unidimensionality of the dataset of each sample was also evaluated considering the Unidimensional Congruence (UniCo), Explained Common Variance (ECV) and Mean of Item Residual Absolute Loadings (MIREAL) indices [27] obtained by the Factor program (V11.0427) [28]. Values of UniCo > 0.95, ECV > 0.85 and MIREAL < 0.30 suggested that the items can be treated as components of a single dimension [27].

After fitting the models to the data, the measurement invariance of the tested models was verified between the samples of each country. When observing the configural invariance of the factorial models between the different samples (Sample 1 vs. 2, Sample 1 vs. 3, Sample 2 vs. 3, and Sample 4 vs. 5), the measure invariance was estimated by multigroup analysis using the CFI difference (ΔCFI). The WLSMV estimation method was used considering ΔCFI between the configural (M0) and metric (M1) models (ΔCFI_M1-M0_) and between the metric and scalar (M2) models (ΔCFI_M2-M1_). A decrease in CFI (ΔCFI) above 0.01 was considered indicative of the absence of measurement invariance [23,29].

### 2.7. Validity Based on Response Process

The validity based on responses process was initially evaluated considering the following item fit statistics: information-weighted mean square (INFIT: people with a latent trait level equivalent to the item difficulty do not respond as expected) and unweighted mean square (OUTFIT: people with a latent trait level different from the item difficulty do not respond as expected). Both statistics were estimated for each sample considering the partial-credit model (PCM) and using the eRm package [30] in the R program (R Core Team, 2020). Values of INFIT and OUTFIT between 0.5 and 1.5 were indicative of an adequate fit of the item to the PCM, being considered productive for measurement.

Then, Differential Item Function analysis (DIF) was conducted between samples of each country (Samples: 1 vs. 2, 1 vs. 3, 2 vs. 3, and 4 vs. 5). For this purpose, ordinal logistic regression was performed based on the likelihood ratio chi-square statistics, considering a significance level of 1%. DIF can be classified as uniform (if the effect is constant) or non-uniform (if the effect varies), and, in this study, a general test of “total DIF effect” was used in order to maximize the capacity of the identification of both uniform and non-uniform DIF and to control Type I error [31]. DIF was performed using the *lordif* package [31] in the R program (R Core Team, 2020) and the items that presented a significant “total DIF effect” (*p* < 0.01) were considered non-equivalent [31].

### 2.8. Consequence Validity

Considering the results obtained in the previous analyses, the ethical consequences and quality of the measures obtained from the use of OHIP-14 in different study samples (dental patient and non-dental patient) were evaluated.

## 3. Results

### 3.1. Content Validity

In both studies, the panel of expert judges considered that the grammatical, semantic and idiomatic terms of the OHIP-14 versions (in Portuguese and Finnish) are clear for the participants’ understanding and that the content of the items is pertinent and relevant for the assessment of the impact of a problem related to oral health on an individual’s life. In Study 1, 57 Brazilians participated in the pilot study (81.0% women, mean age: 28.4 (standard deviation = 5.5) years) and in Study 2, 37 Finns (67.6% women, mean age: 31.2 (standard deviation = 11.0) years). In Brazil, the OHIP-14 items presented an Incomprehension Index between 0.0% and 3.5% and in Finland this index was between 0.0% and 5.4%. Therefore, the understanding of the items by the participants was considered adequate, attesting the content validity of OHIP-14 in both countries.

### 3.2. Validity Based on Internal Structure

The summary measures of the responses given by the participants are shown in Table 2. The responses from non-dental patient samples from both countries (Samples 2 and 5) showed high values of skewness and kurtosis in six items of the OHIP-14. In Brazil, items 1, 2, 11, 12, 13, and 14, and in Finland items 1, 2, 7, 11, 12, and 14, indicated severe violation of the normal distribution. Thus, as these items did not meet a relevant assumption, they could not be included in subsequent analyses to assess validity based on the internal structure. As the number of items that violated this assumption represents 42.9% of the items in the OHIP-14, a decision was made to not proceed with the analyses for these samples.

Regarding the samples of dental patients, all the responses given to the OHIP-14 items by the participants in Sample 3 (Brazil) presented adequate values for skewness and kurtosis. For Sample 1 (Brazil) and Sample 4 (Finland), a severe violation of the normal distribution was observed in only one item (Brazil: item 14 “unable to function”, Finland: item 2 “taste worse”). Thus, the item whose responses did not meet the assumption was not considered for the subsequent analyses.

Concerning the factorial models referring to the original OHIP-14 proposal (Figure 1a–c), due to the need to exclude the item that violated the assumption of normality in Sample 1 and Sample 4 (items 14 and 2, respectively), it was also necessary to exclude the factor where the items were allocated (Sample 2: “Handicap”; Sample 4: “Functional Limitation”), since in these models each first-order factor is composed of only two items. The covariance matrix of the first-order factorial proposal of the OHIP-14 (Figure 1a) was not defined as positive for the tested samples (Samples 1, 3 and 4), indicating that this model does not present an adequate fit to the data.

The second- and third-order hierarchical models containing seven first-order factors (Figure 1b,c) presented an adequate fit to the Sample 3 data only after restricting the variance of some factors (Table 3), suggesting caution in interpreting these results. For samples 1 and 4, these hierarchical models were not tested, because, given the exclusion of a first-order factor, there is no theoretical plausibility for the elaboration of these models.

The models containing three first-order factors (Figure 1e,f) showed an adequate fit to the samples of Brazilian dental patients (Table 3, Samples 1 and 3) and the convergent validity and reliability were adequate. Discriminant validity had limitations in both samples. For Sample 4, in addition to item 2, it was also necessary to exclude item 1 and the “Functional Limitation” factor for the model to present an adequate fit. This model also had convergent and discriminant validity and adequate reliability for the data in Sample 4 (Table 3). The hierarchical model was not tested for this sample, since the factorial model now has two first-order factors, with no theoretical and analytical plausibility for the inclusion of a second-order factor.

In relation to the unifactorial model (Table 3), RMSEA values above 0.10 in Samples 2 and 4 were observed. This index is overestimated in simple factorial models [32]; therefore, the SRMR is an alternative for decision-making regarding the fit of the model [32]. In addition to an adequate fit to the data, the unifactorial model showed adequate convergent validity and reliability in samples of dental patients from both countries. Furthermore, values of UniCo ≥ 0.98, ECV ≥ 0.87 and MIREAL ≤ 0.23 were observed in these samples, indicating that the data obtained from samples of dental patients can be treated as unidimensional.

In the samples of Brazilian dental patients (Study 1, Samples 1 and 3), strong measure invariance was observed in the factorial model with three first-order factors (ΔCFI_M1-M0_ = −0.007, ΔCFI_M2-M1_ = −0.007) and in the unifactorial model (ΔCFI_M1-M0_ = −0.007, ΔCFI_M2-M1_ = −0.010). It is noteworthy that, to enable the invariance analysis, the OHIP-14 was considered without item 14 to establish configural invariance. This strategy was used to present an analysis related to the stability of measurement functioning between independent samples. The measurement invariance was not tested between the samples of the non-dental patients and dental patients from both countries. As shown above, more than 40% of the items did not present psychometric sensitivity in the samples of the non-dental patients (Sample 2 and Sample 5) and it was not possible to fit the factorial models to the data in these samples. This result is an indication that the OHIP-14 works differently between the samples and that it does not seem to be an adequate instrument to identify the construct of the oral health impact profile on life in non-dental patient samples.

### 3.3. Validity Based on Response Process

**Figure 2:**
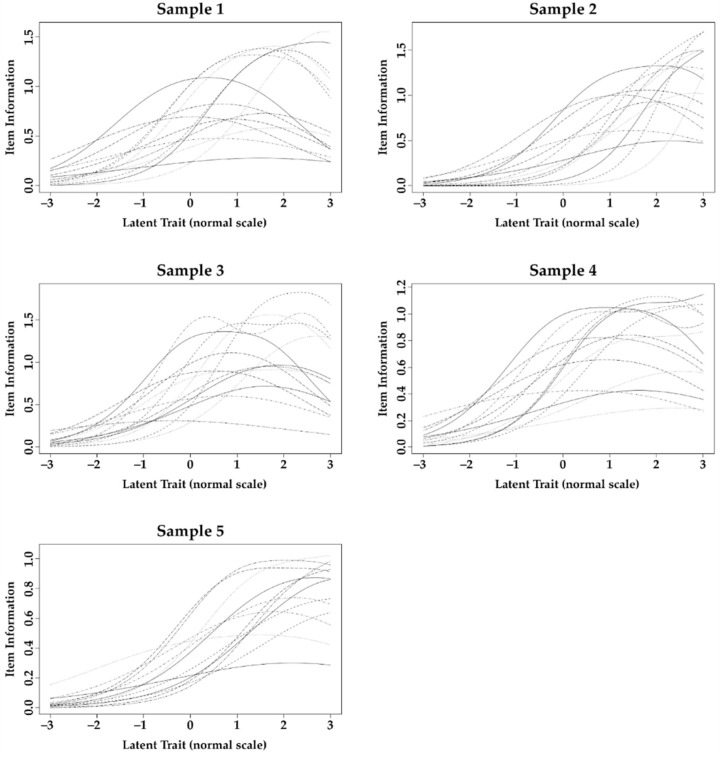
Item information function obtained from Differential Item Functioning analysis of the items of Oral Health Impact Profile-14 applied to the samples of the studies.

Table 4 presents item fit statistics. It is observed that, in general, the OHIP-14 items present difficulties compatible with the latent trait of each one of the samples. However, the results of DIF analysis (Table 4) indicate that at least one item was responded to significantly differently between the study samples. This difference can also be observed by the item information function in the samples (Figure 2). In general, the OHIP-14 items are informative for different levels (mild to severe) of impact of oral health on life for dental patient samples only (Sample 1, Sample 3 and Sample 4; latent trait ≤ 2 responds from 57.9% to 74.4% of the amount of information obtained by the instrument), while for the non-dental patient samples (Sample 2 and Sample 5) they are informative only when extremely severe levels of impact are present (latent trait ≥ 2 responds de 59.1% to 60.7% of the amount of information obtained by the instrument).

### 3.4. Consequence Validity

The evidence obtained in both studies supports consequence validity, pointing out the consequences of using the OHIP-14 in different contexts in which the instrument was developed for application. It is important to emphasize that a psychometric instrument, such as the OHIP-14, is elaborated to measure a dimension (construct) in a specific population context. Thus, its use in a different population context from the one originally proposed needs to be previously evaluated, as the results obtained may have consequences both for an individual’s life and for the definition of a treatment plan and/or clinical follow-up. Thereby, the evidence presented in Studies 1 and 2 indicates an ethical concern in obtaining and using the OHIP-14 measure and supports the non-indication of using this instrument in individuals or samples without impairment of oral health.

## 4. Discussion

This study presented results regarding how OHIP-14 works in samples of dental patients and non-dental patients in two different countries. Although previous studies have sought information regarding both the dimensionality (factorial structure) of the OHIP and its fit to data from different samples [2,13,15,16,18], this topic is still relevant for the following reasons. First, despite wide use of the OHIP-14, evaluation of the validity of the data obtained has often been neglected. As OHIP-14 is a psychometric instrument, it is necessary to ensure that it measures what it proposes to measure when applied to different samples and/or contexts. This assessment is made through validity analyses, as proposed in the Standards for Educational and Psychological Testing [21]. Second, validity does not refer to the instrument itself, but to the data obtained from its application in a specific sample and context. Therefore, obtaining evidence on how the instrument works in different samples can contribute to the decision to choose the OHIP-14 as a measurement instrument, whether for research or clinical purposes.

The study revealed that the OHIP-14 did not work properly in samples of the non-dental patients in either Brazil or Finland. In other words, OHIP-14 did not adequately measure the profile of the impact of oral health on an individual’s life. This differs from the results presented by Montero et al. [13], who found an adequate fit of the trifactorial model to a Spanish sample of non-dental patients. The difference can be attributed both to the impact of oral health on life being perceived differently between countries, as well as to the analytical strategy used in the studies. Despite using confirmatory factor analysis to verify the fit of the OHIP-14 to the data, Montero et al. [13] used the Maximum Likelihood (ML) estimation method, which assumes that the data have normal distribution. However, meeting this assumption was not mentioned, limiting the possibility to verify the fit of the model to the data. In the present study, we adopted the robust estimation method (WLSMV), indicated for an ordinal response scale (such as that of the OHIP-14) and a set of responses with a slight violation of the assumption of normal distribution [22]. However, 6 items of the OHIP showed severe violation of normal distribution in samples of the non-dental patients both in Brazil and Finland, limiting the confirmatory analysis, even using the robust estimator. Furthermore, this severe violation of normality would imply the need to exclude approximately 43% of the instrument’s items since they do not adequately capture the variability of responses in this population. This indicates that the OHIP-14 does not measure properly what it is proposed to measure in these samples. This may have occurred since the content of these items refers to orofacial problems, which seem to be less prevalent in non-dental patients. Therefore, the application of OHIP-14 in non-dental patient samples deserves attention as it may not measure the intended OHRQoL-related dimension; thus, the obtained result may not represent reality and lead to erroneous conclusions for these samples.

For dental patient samples, the OHIP-14 presented adequate fit and measurement validity. However, it was necessary to verify which factor structure proposed in the literature fitted properly to the present data. The structure based on Locker’s theoretical model [9] on 7 first-order factors was tested first. This structure presented adequate fit for only one of the datasets of dental patient samples (Sample 3) and this was only possible after the inclusion of hierarchical factors and variance restriction. The difficulty of fitting this model was previously observed by Baker et al. [19], who suggest that this structure may contain overlapping concepts, making its fit difficult. Furthermore, Baker et al. [19] suggested that the dimensions presented do not necessarily reflect the content of the items, since the dimensions were elaborated based on the 1980 WHO Classification of Impairments, Disabilities, and Handicaps model [33], which was no longer valid after 2001 [3]. Thus, it is essential to identify the dimensionality of OHIP-14, as well as its underlying theoretical concept, to ensure the validity of data obtained in new samples and current contexts.

Montero et al. [13] found a trifactorial structure (Psychosocial Impacts, Pain-Discomfort, and Functional Limitation) of the OHIP-14 using exploratory factor analysis. This structure seems interesting because it allows the identification of three dimensions that are present in the most current theoretical proposal elaborated by John [3,34]. In this new perspective, the OHRQoL is structured from the dimensions Psychosocial Impact, Orofacial Pain, Oral Function, and Orofacial Appearance, which represent the reasons that lead an individual to seek dental treatment. When the trifactorial model was tested, we observed an adequate fit to the data from Brazilian dental patient samples. For the Finnish dental patient sample, adequate fit for this proposal was only achieved after eliminating the Functional Limitation dimension. These findings show that the theoretical proposal of OHIP-14 as a trifactorial model is plausible for measuring the different dimensions of OHRQoL in dental patient samples. We also emphasize that, if the researcher/professional aims to assess the OHRQoL in a broader way, seeking to contemplate the four dimensions proposed by John [3,34], an investigation protocol that includes other additional psychometric instruments and/or that uses a more comprehensive instrument is necessary, such as the OHIP-49, as the OHIP-14 cannot fully cover this theoretical model.

The unifactorial model of the OHIP-14 was proposed by Santos et al. [16] In the present study, the unifactorial model presented an adequate fit to data from dental patient samples, both in Brazil and Finland. It was also observed that the unidimensionality indices (UniCo, ECV and MIREAL) suggest that, for these samples, although the trifactorial model presented adequate fit, the unifactorial model is more interesting. Thus, the unifactorial model was the one that was best applied to measure the oral health impact profile on the lives of participants in the present study samples.

In view of the results and what was revealed in the introduction of this study, those who choose to use the OHIP-14, whether for clinical or research purposes, are cautioned to be mindful of the way in which the OHIP-14 results are interpreted. Some studies refer to the construct assessed as quality of life or OHRQoL in general, but what is actually measured is the perception of the impact of a given oral condition on an individual’s life [18], which can be considered as a single construct (unifactorial model) or from different dimensions (trifactorial model). Thus, it is only one of the components of what is considered quality of life, which in turn is a complex and multidimensional concept that includes components, for example, related to good living conditions, life functionality and the ability to cope with life’s challenges [35]. Therefore, OHIP-14 results as a sole measure, without considering population characteristics (nationality, age, etc.) and oral condition, may be difficult to be interpreted. Those results should be considered in addition to the clinical findings, providing the professional with important information regarding the patient’s perspective regarding the impact of the oral condition on their life [3,34]. With this information in hand, the professional will be able to move towards the real demands and expectations of patients, placing them in the central role in the elaboration of treatment.

Another point to note is that the OHIP, whether in its complete or reduced version, was elaborated more than two decades ago, having its theoretical structure based on a model (WHO Classification of Impairments, Disabilities, and Handicaps [33]) that is no longer applied. Despite the undeniable usefulness of the OHIP nowadays, it is necessary to take a critical look at how much the items in this instrument really fit into different samples and current contexts. Based on the present study, it is not recommended its use for data collection without prior evaluation of the validity of the obtained data. It can be recommended to ensure how OHIP-14, or any psychometric instrument [21], works when applied to a specific population and in a new context.

The results of this study corroborate the above issues since the way OHIP-14 works was affected by the characteristics of the target population and was not fitted for the non-dental patients. Furthermore, although we have considered dental patient samples regardless of oral condition and treatment, specific groups of these patients can also affect how OHIP-14 works. For example, the OHIP-14 may not work properly for a group of dental patients with demand exclusively related to orofacial appearance, as the content of the items in this instrument is more related to the other dimensions of the OHRQoL (Psychosocial Impact, Orofacial Pain and Oral Function). For clinicians, the choice of an instrument should be based on this evidence obtained in a population with characteristics similar to those of their patients, otherwise the result attributed to the instrument answered by the patient may be arbitrary, which will negatively influence the plan of treatment, where under or over treatment may occur.

Convenience sampling can be considered as a limitation of this study. This type of sampling is, however, commonly used in observational studies that aim to estimate the psychometric properties of measurement instruments in different samples/contexts [10,13,15,18]. The use of a sample of dental patients in general, without specifying a clinical condition, can also be considered a limitation since, as previously mentioned, the clinical condition can affect how the instrument works. However, we clarify that our aim with this study was to open a deeper discussion about what we are measuring with the OHIP-14 and highlighting, as a starting point, the comparison between dental patient and non-dental patient samples. Future investigations that conduct studies in populations with specific clinical conditions are of interest to verify the impact that each one of these can have on the way to measure the oral health impact construct on patients’ lives. It is hoped that the results obtained and discussions raised in this study can serve as a basis for reflections for these new studies.

Despite these limitations, consistent evidence was presented in this study regarding the non-operationalization of the OHIP-14 in non-dental patient samples in Brazil and Finland and the possible ways in which it works in dental patient samples from both countries. Thus, it is expected that the study will contribute to the advancement of Evidence-Based Dentistry, as it provides information to alert researchers to the responsibility and need to obtain valid data with adequate interpretation when using OHIP-14 in different samples. For clinicians, we emphasize the need to choose and use the best way to gather information that may be relevant to the development of a patient-centered treatment plan.

## 5. Conclusions

The validity of data related to the impact of oral health problems on individuals’ lives was confirmed through a unifactorial model. OHIP-14 works properly in dental patient samples and was limited in non-dental patient samples and was also influenced by cultural context and age.

## Figures and Tables

**Table 1:** Description of study samples.

	Study 1—Brazil			Study 2—Finland	
	Sample 1	Sample 2	Sample 3	Sample 4	Sample 5
Population	Dental patients	Non-dental patients	Dental patients (Zucoloto et al. [18])	Dental patients	Non-dental patients
Year of data collection	2018–2019	2018–2019	2012–2013	2020	2020
Collection method	paper-and-pencil	paper-and-pencil	paper-and-pencil	online	online
n	434	1486	439	482	2425
% women	76.5	67.9	74.0	80.7	75.0
Mean age (standard deviation) in years	25.3 (6.3)	24.7 (5.6)	29.0 (6.7)	26.3 (5.4)	26.7 (5.5)

**Table 2:** Descriptive statistics of the responses given to the items of the Oral Health Impact Profile-14 by the participants of each study.

Study 1—Brazil (Sample 1/Sample 2/Sample 3) *							
**Item**	**Mean**	**Median**	**Standard Deviation**	**Minimum**	**Maximum**	**Skewness**	**Kurtosis**
It1	0.50/0.20/0.54	0/0/0	0.94/0.60/1.05	0/0/0	4/4/4	2.06/3.74/1.96	3.75/16.11/2.98
It2	0.43/0.21/0.73	0/0/0	0.87/0.61/1.20	0/0/0	4/4/4	2.13/3.48/1.46	3.97/13.41/0.95
It3	1.45/0.91/1.38	1/1/1	1.15/0.98/1.17	0/0/0	4/4/4	0.43/0.85/0.51	−0.54/0.06/−0.41
It4	1.47/0.77/1.59	1/0/2	1.26/1.04/1.32	0/0/0	4/4/4	0.47/1.24/0.33	−0.74/0.74/−0.91
It5	2.03/1.12/2.42	2/1/2	1.23/1.19/1.35	0/0/0	4/4/4	0.12/0.84/−0.31	−0.87/−0.21/−1.01
It6	1.71/0.79/1.39	2/0/1	1.35/1.10/1.41	0/0/0	4/4/4	0.25/1.31/0.55	−1.09/0.85/−0.98
It7	1.02/0.35/0.90	1/0/0	1.25/0.79/1.20	0/0/0	4/4/4	1.05/2.63/1.14	0.02/7.09/0.24
It8	0.86/0.28/1.05	0/0/1	1.17/0.70/1.11	0/0/0	4/4/4	1.26/2.94/0.75	0.63/9.35/−0.16
It9	0.99/0.44/1.13	0/0/1	1.22/0.85/1.29	0/0/0	4/4/4	1.00/2.12/0.82	−0.07/4.18/−0.42
It10	1.07/0.48/1.25	1/0/1	1.32/0.94/1.38	0/0/0	4/4/4	0.99/2.15/0.71	−0.24/4.11/−0.74
It11	0.62/0.26/0.70	0/0/0	1.04/0.72/1.05	0/0/0	4/4/4	1.79/3.28/1.42	2.48/11.12/1.33
It12	0.54/0.20/0.58	0/0/0	0.94/0.60/0.97	0/0/0	4/4/4	1.85/3.58/1.73	2.93/14.62/2.44
It13	0.57/0.21/0.58	0/0/0	1.04/0.64/1.10	0/0/0	4/4/4	1.89/3.69/1.90	2.82/14.85/2.67
It14	0.26/0.09/0.28	0/0/0	0.74/0.46/0.75	0/0/0	4/4/4	3.43/6.08/2.99	12.34/41.42/9.22
Study 2—Finland (Sample 4/Sample 5) *							
**Item**	**Mean**	**Median**	**Standard Deviation**	**Minimum**	**Maximum**	**Skewness**	**Kurtosis**
It1	0.51/0.19	0/0	0.94/0.58	0/0	4/4	1.75/3.57	2.09/14.40
It2	0.14/0.05	0/0	0.48/0.26	0/0	4/3	4.32/6.18	22.99/42.61
It3	1.84/1.27	2/1	0.86/0.83	0/0	4/4	0.25/0.30	0.40/−0.04
It4	1.36/0.66	1/0	1.09/0.89	0/0	4/4	0.41/1.29	−0.42/1.12
It5	1.31/0.67	1/0	1.11/0.92	0/0	4/4	0.42/1.21	−0.65/0.70
It6	1.28/0.63	1/0	1.20/0.91	0/0	4/4	0.56/1.35	−0.64/1.10
It7	0.39/0.12	0/0	0.75/0.44	0/0	4/4	2.08/4.31	4.35/22.07
It8	0.53/0.20	0/0	0.79/0.50	0/0	4/4	1.38/2.85	1.38/9.20
It9	0.99/0.42	1/0	1.08/0.75	0/0	4/4	0.86/1.87	0.00/3.32
It10	0.87/0.43	0/0	1.08/0.77	0/0	4/4	1.03/1.84	0.16/2.99
It11	0.57/0.18	0/0	0.84/0.49	0/0	4/4	1.35/3.06	1.15/10.50
It12	0.56/0.15	0/0	0.86/0.45	0/0	4/4	1.56/3.43	2.06/14.08
It13	0.78/0.35	0/0	1.03/0.69	0/0	4/4	1.29/2.15	1.03/4.60
It14	0.22/0.05	0/0	0.57/0.28	0/0	4/4	2.92/6.91	9.22/60.01

Note: Slash punctuation marks were inserted between the estimates to separate the values obtained for each sample. * Sample 1: Brazil, dental patient, paper-and-pencil; Sample 2: Brazil, non-dental patient, paper-and-pencil; Sample 3: Brazil, dental patient, paper-and-pencil; Sample 4: Finland, dental patient, online; Sample 5: Finland, non-dental patient, online.

**Table 3:** Fit of factorial models of the Oral Health Impact Profile-14 to data from different samples.

				CFA ^#^											
Model	Sample *	Excluded Items	n	CFI	TLI	RMSEA	SRMR	λ	r^2^	β-2nd Order	β-3rd Order	α ^†^	CR ^¶^	AVE ^§^	Observation
7 Factors—2nd Order	Sample 3	-	439	0.985	0.980	0.064	0.046	0.58–0.96	0.77–0.85	0.88–0.99	-	0.72–0.85	0.73–0.87	0.57–0.77	Factors with restriction on error variance: Psychological Disability e Handicap
7 Factors—3rd Order	Sample 3	-	439	0.985	0.980	0.064	0.046	0.58–0.96	-	0.88–0.99	0.93–0.98	0.72–0.85	0.73–0.87	0.57–0.77	Factors with restriction on error variance: Psychological Disability e Handicap
3 Factors—1st Order	Sample 1	14	434	0.947	0.933	0.115	0.065	0.63–0.88	0.43–0.73	-	-	0.69–0.91	0.70–0.92	0.55–0.64	-
	Sample 3	-	439	0.980	0.976	0.071	0.053	0.53–0.90	0.78–0.86	-	-	0.76–0.92	0.77–0.93	0.62–0.64	-
	Sample 3	14 ^‡^	439	0.983	0.979	0.069	0.047	0.54–0.90	0.77–0.85	-	-	0.76–0.91	0.77–0.92	0.61–0.64	-
	Sample 4	1 and 2	482	0.972	0.965	0.098	0.064	0.64–0.89	0.63	-	-	0.87–0.93	0.88–0.94	0.64–0.65	Excluded factor: Functional Limitation
3 Factors—2nd Order	Sample 1	14	434	0.947	0.933	0.115	0.065	0.63–0.88	-	0.75–0.98	-	0.69–0.91	0.70–0.92	0.55–0.64	-
	Sample 3	-	439	0.980	0.976	0.071	0.053	0.53–0.90	-	0.93–0.97	-	0.76–0.92	0.77–0.93	0.62–0.64	-
	Sample 3	14 ^‡^	439	0.983	0.979	0.069	0.047	0.54–0.90	-	0.93–0.97	-	0.76–0.91	0.77–0.92	0.61–0.64	-
Unifactorial	Sample 1	14	434	0.925	0.910	0.134	0.077	0.47–0.87	-	-	-	0.93	0.94	0.55	-
	Sample 3	-	439	0.972	0.967	0.082	0.059	0.52–0.86	-	-	-	0.95	0.95	0.59	-
	Sample 3	14 ^‡^	439	0.975	0.970	0.082	0.053	0.53–0.87	-	-	-	0.94	0.95	0.58	-
	Sample 4	2	482	0.949	0.938	0.120	0.078	0.54–0.85	-	-	-	0.94	0.95	0.57	-

Note: * Sample 1: Brazil, dental patient, paper-and-pencil; Sample 3: Brazil, dental patient, paper-and-pencil; Sample 4: Finland, dental patient, online. # CFA: confirmatory factor analysis, CFI: comparative fit index, TLI: Tucker-Lewis index, RMSEA: root mean square error of approximation, SRMR: standardized root mean square residual, λ: factorial loading, r^2^: square correlation coefficient between the factors, β: absolute value of β estimate. ^†^ α: ordinal alpha coefficient. ^¶^ CR: composite reliability. ^§^ AVE: average extracted variance. ^‡^ Item excluded to obtain configural invariance between Sample 1 and Sample 3.

**Table 4:** Item fit statistics (information-weighted mean square [INFIT] and unweighted mean square [OUTFIT]) for each sample and Differential Item Functioning (DIF) analysis results between samples.

	Item Fit Statistics										DIF *p*-Value for χ^2^			
	Sample 1		Sample 2		Sample 3		Sample 4		Sample 5		Sample 1 vs. 2	Sample 1 vs. 3	Sample 2 vs. 3	Sample 4 vs. 5
Item	Infit	Outfit	Infit	Outfit	Infit	Outfit	Infit	Outfit	Infit	Outfit				
it1	1.37	1.95	1.44	1.96	1.21	1.99	1.38	1.96	1.27	1.73	0.173	0.007	0.033	0.038
it2	1.20	1.05	1.05	1.28	0.88	0.66	1.16	1.36	1.00	1.17	0.020	<0.001	<0.001	0.408
it3	1.12	1.14	1.16	1.16	1.09	1.15	1.06	1.06	0.96	0.94	0.221	0.197	0.07	<0.001
it4	0.91	0.88	0.92	0.88	0.93	0.93	1.09	1.05	1.09	0.96	0.349	0.789	<0.001	<0.001
it5	0.90	0.90	0.89	0.87	1.36	1.39	0.85	0.83	0.77	0.70	<0.001	<0.001	<0.001	<0.001
it6	0.74	0.69	0.73	0.67	0.77	0.72	0.83	0.77	0.80	0.73	0.068	<0.001	0.485	0.050
it7	0.77	0.69	0.73	0.61	0.74	0.59	0.85	0.62	0.90	0.65	0.033	<0.001	0.011	0.403
it8	0.75	0.75	0.78	0.72	0.84	0.79	0.83	0.73	0.91	0.82	0.028	<0.001	<0.001	0.136
it9	0.76	0.68	0.89	0.86	0.71	0.63	0.78	0.70	0.79	0.61	0.067	0.321	<0.001	0.134
it10	1.21	1.32	1.18	1.26	0.95	1.06	1.00	1.00	0.97	0.90	0.482	0.06	<0.001	0.194
it11	1.11	1.04	0.92	1.03	1.00	0.87	0.75	0.59	0.80	0.48	0.080	0.73	<0.001	0.168
it12	0.74	0.70	0.76	0.62	0.97	0.86	0.74	0.74	0.79	0.51	0.564	0.22	<0.001	0.001
it13	0.86	0.70	0.70	0.41	0.77	0.86	0.78	0.85	0.89	0.79	0.395	0.027	0.903	0.642
it14	0.81	0.58	0.85	0.37	0.91	0.56	1.03	0.97	0.98	1.04	0.293	0.778	0.085	0.015

Note: Sample 1: Brazil, dental patient, paper-and-pencil; Sample 2: Brazil, non-dental patient, paper-and-pencil; Sample 3: Brazil, dental patient, paper-and-pencil; Sample 4: Finland, dental patient, online; Sample 5: Finland, non-dental patient, online.

## Data Availability

Not applicable.
